# Palmitic Acid Induces Osteoblastic Differentiation in Vascular Smooth Muscle Cells through ACSL3 and NF-κB, Novel Targets of Eicosapentaenoic Acid

**DOI:** 10.1371/journal.pone.0068197

**Published:** 2013-06-28

**Authors:** Aiko Kageyama, Hiroki Matsui, Masahiko Ohta, Keisuke Sambuichi, Hiroyuki Kawano, Tatsuto Notsu, Kazunori Imada, Tomoyuki Yokoyama, Masahiko Kurabayashi

**Affiliations:** 1 Development Research, Mochida Pharmaceutical Co., Ltd., Gotemba, Shizuoka, Japan; 2 Department of Medicine and Biological Science, Gunma University Graduate School of Medicine, Maebashi, Gunma, Japan; 3 Department of Laboratory Sciences, Gunma University Graduate School of Health Sciences, Maebashi, Gunma, Japan; UAE University, Faculty of Medicine & Health Sciences, United Arab Emirates

## Abstract

Free fatty acids (FFAs), elevated in metabolic syndrome and diabetes, play a crucial role in the development of atherosclerotic cardiovascular disease, and eicosapentaenoic acid (EPA) counteracts many aspects of FFA-induced vascular pathology. Although vascular calcification is invariably associated with atherosclerosis, the mechanisms involved are not completely elucidated. In this study, we tested the hypothesis that EPA prevents the osteoblastic differentiation and mineralization of vascular smooth muscle cells (VSMC) induced by palmitic acid (PA), the most abundant long-chain saturated fatty acid in plasma. PA increased and EPA abolished the expression of the genes for bone-related proteins, including bone morphogenetic protein (BMP)-2, Msx2 and osteopontin in human aortic smooth muscle cells (HASMC). Among the long-chain acyl-CoA synthetase (ACSL) subfamily, ACSL3 expression was predominant in HASMC, and PA robustly increased and EPA efficiently inhibited ACSL3 expression. Importantly, PA-induced osteoblastic differentiation was mediated, at least in part, by ACSL3 activation because acyl-CoA synthetase (ACS) inhibitor or siRNA targeted to ACSL3 completely prevented the PA induction of both BMP-2 and Msx2. Conversely, adenovirus-mediated ACSL3 overexpression enhanced PA-induced BMP-2 and Msx2 expression. In addition, EPA, ACSL3 siRNA and ACS inhibitor attenuated calcium deposition and caspase activation induced by PA. Notably, PA induced activation of NF-κB, and NF-κB inhibitor prevented PA-induction of osteoblastic gene expression and calcium deposition. Immunohistochemistry revealed the prominent expression of ACSL3 in VSMC and macrophages in human non-calcifying and calcifying atherosclerotic plaques from the carotid arteries. These results identify ACSL3 and NF-κB as mediators of PA-induced osteoblastic differentiation and calcium deposition in VSMC and suggest that EPA prevents vascular calcification by inhibiting such a new molecular pathway elicited by PA.

## Introduction

Vascular calcification commonly occurs with advancing age, chronic kidney disease, diabetes mellitus and atherosclerosis, and is closely associated with cardiovascular morbidity and mortality [1]–[3]. Vascular calcification has long been simply viewed as the final stage of degeneration and necrosis of arterial wall and a passive, unregulated process. However, accumulating evidence points toward an active and tightly regulated process that resembles bone mineralization, with phenotypic transition of vascular smooth muscle cells (VSMC). Two pathophysiological processes, osteoblastic differentiation and apoptosis, are involved in the development of vascular calcification [4]. Osteoblastic differentiation of VSMC is characterized by the expression of bone-related molecules including bone morphogenetic protein (BMP)-2, Msx2 and osteopontin, which are produced by osteoblasts and chondrocytes and are observed in calcified lesions [5], [6].

Plasma concentrations of free fatty acids (FFAs) are increased in patients with metabolic syndrome [7], obesity [8] and type 2 diabetes mellitus [9]. Specifically, saturated FFAs significantly contribute to the development of atherosclerosis [10]. Increasing evidence indicates that saturated FFAs activate inflammatory signaling pathways in vascular cells, including VSMC, macrophages and vascular endothelial cells. Furthermore, Miyazaki and his colleagues have provided evidence indicating that liver X receptor (LXR)-induced lipogenesis and saturated fatty acids stearic acid (SA) induce vascular calcification *in vitro* [11], [12].

Increasing numbers of clinical and experimental studies, especially prospective randomized clinical trials, have demonstrated the beneficial effects of n-3 polyunsaturated fatty acids (PUFAs). The most compelling evidence for the cardiovascular benefits of n-3 PUFAs comes from studies of primary and especially secondary prevention of coronary heart disease, and most recently, in post-myocardial infarction patients with heart failure. In Japan EPA Lipid Intervention Study (JELIS) trial, patients randomized to eicosapentaenoic acid (EPA), a major n-3 PUFA contained in fish oil, had a 19% reduction in major cardiovascular events [13]. There have been some reports describing the inhibitory effects of EPA or n-3 PUFAs on ectopic calcification. N-3 PUFAs supplementation has been described to reduce ectopic calcification *in vivo* [14], [15]. Two recent studies show that EPA inhibits warfarin-induced arterial medial calcification in rats [16], and mineralization of calcifying vascular cells (CVC) *in vitro* [17]. Recently, a clinical study (Rotterdam study) revealed a weak inverse association between fish intake and coronary calcification [18].

Among FFAs, saturated fatty acids such as palmitic acid (PA) have been shown to induce lipotoxicity in several types of cells including endothelial cells [19], [20] and cardiac myocytes [21]. Elevated levels of FFAs play an important role in the pathogenesis of metabolic diseases including atherosclerosis [22], [23] and type 2 diabetes [24]. A variety of chemoattractant and inflammatory mediators including tumor necrosis factor α, interleukin-1β, monocyte chemoattractant protein-1, leptin and FFA *per se*, are synthesized and secreted from adipose tissue and macrophages when FFAs are abundantly accumulated in the adipocytes and adipose tissue macrophages. Those adipose tissue-derived mediators have been implicated in the endothelial dysfunction, macrophage activation and VSMC proliferation in the vessel wall, which play a prominent role in the formation of atherosclerotic plaque [25]. In addition to these systemic responses, PA gives rise to a distinct impact on the metabolism within the vessel wall; PA directly induces endothelial dysfunction and/or apoptosis through a generation of oxidative stress [26], [27]. On the other hand, EPA prevents PA-induced lipotoxicity in pancreatic β-cells [28] and inflammatory change in macrophages [29]. However, the effects of EPA on vascular calcification induced by saturated fatty acids have not been determined.

In this study, we examined whether PA induces osteoblastic differentiation and calcium deposition in human aortic smooth muscle cells (HASMC). Our findings showed that PA promotes both osteoblastic differentiation of VSMC, as determined by the expression of osteoblastic genes such as BMP-2, Msx2 and osteopontin, and calcium deposition mediated by apoptosis. We also investigated the mechanisms of PA-induced osteoblastic differentiation of HASMC and the effect of EPA on osteoblastic differentiation induced by PA. Our findings indicated that long-chain acyl-CoA synthetase (ACSL) 3- and NF-κB-dependent pathways mediate PA-induced osteoblastic differentiation and calcium deposition in VSMC, and that EPA prevents these changes at least in part by interfering with PA-induced ACSL3 expression.

## Materials and Methods

### Cell Culture for Osteoblastic Differentiation

Primary HASMC were purchased from Kurabo (Osaka, Japan) and maintained in Humedia-SG2 (Humedia-SB2 supplemented with 5% FBS, 0.5 ng/mL hEGF, 2.0 ng/mL hFGF-B, 5.0 µg/mL insulin, 50 µg/mL gentamicin and 50 ng/mL amphotericin B) (Kurabo) at 37°C in a humidified atmosphere with 5% CO_2_.

HASMC were seeded at a density of 2.3×10^4^ cells/cm^2^ in collagen-coated plates and allowed to attach overnight. Subsequently, the standard culture medium was replaced by Humedia-SB2 (Kurabo) supplemented with 0.1% BSA, 1% PBS and antibiotics. The following day, HASMC were exposed to 250 µM PA (Sigma-Aldrich, St Louis, MO) in the presence or absence of 15 µM EPA (Nu-Chek Prep, Elysian, MN), 2 µM triacsin C (acyl-CoA synthetase [ACS] inhibitor, Alexis, Plymouth Meeting, PA), 100 µM hypoestoxide (IκB kinase [IKK] inhibitor, Calbiochem, San Diego, CA) in Humedia-SB2 supplemented with 0.3% BSA, 3% PBS and antibiotics for 1 day. After treatment, HASMC were harvested for quantitative real-time PCR, Western blot analysis and measurement of ACS activity.

For knock-down experiments, small interfering RNAs (siRNA) targeted at human ACSL3 (Stealth™ Select RNAi, Oligo ID: HSS176683) were obtained from Invitrogen (Carlsbad, CA), using sense 5′-UAAUCUUUAAGCAUCCAUCGGGUUC-3′ and antisense 5′- GAACCCGAUGGAUGCUUAAAGAUUA-3′ primers. HASMC were transfected with siRNA in a final concentration of 50 nM using Lipofectamine™ RNAiMAX Reagent (Invitrogen) after several hours of seeding. Stealth™ RNAi Negative Control (Invitrogen) with a similar GC content was used as a control. One day after transfection, medium was replaced by Humedia-SB2 supplemented with 0.1% BSA and 1% PBS. The following day, HASMC were treated with PA as described above.

### Cell Culture for Calcium Deposition

For experiments related to calcium deposition and caspase activation, HASMC were grown until confluent in Humedia-SG2 medium, and then treated with 1 mM PA in the presence or absence of 15 µM EPA, 2 µM triacsin C, 100 µM hypoestoxide and 100 µM Z-VAD-FMK (Calbiochem) in Humedia-SG2 medium containing with 0.3% BSA and 3% PBS for 1 day. After treatment, HASMC were harvested for measurements of calcium deposition and caspase activity.

For knock-down of ACSL3, HASMC were treated with ACSL3 siRNA as described above at 1 day before treatment with PA.

### RNA Isolation and Quantitative Real-Time PCR

Total RNA was isolated from HASMC using the RNeasy® Mini Kit (Qiagen, Valencia, CA) according to the manufacturer’s protocol. Complementary DNA was generated using SuperScript™ III First-Strand Synthesis System (Invitrogen). Quantitative real-time PCR was performed using the ABI Prism® 7000 (Applied Biosystems, Foster City, CA). Gene expression levels were measured using the TaqMan® Gene Expression Assays (Applied Biosystems) for each gene: BMP-2 (Hs00154192_m1), Msx2 (Hs00741177_m1), osteopontin (Hs00167093_m1), ACSL1 (Hs00960561_m1), ACSL3 (Hs00244853_m1), ACSL4 (Hs00244871_m1), ACSL5 (Hs00212106_m1) and ACSL6 (Hs00362960_m1). To normalize expression data, GAPDH gene expression was measured using the Pre-Developed TaqMan® Assay Reagent for human GAPDH (Applied Biosystems) as an internal control.

### Analysis of ACS Activity

ACS activity was measured *via* a modified protocol described by Askari et al. [30]. HASMC were homogenized with ice-cold cell lysis buffer containing 50 mM potassium phosphate (pH 7.4), 10% glycerol, 1 mM EDTA, 20 µg/mL leupeptin, 5 µg/mL pepstatin, 10 µg/mL aprotinin and 5 mM benzamidine (100 µL/2 wells in 96-well plates). The assay contained 10 mM ATP, 250 µM CoA, 175 mM Tris, 5 mM DTT, 8 mM MgCl_2_, 50 µM PA and [1-^14^C] PA (9.25 kBq, Moravek Biochemicals, Brea, CA) in a total volume of 250 µL. The reaction was initiated with the addition of cell lysates (50–60 µL/assay), followed by an incubation period of 40 min at 37°C. The reaction was stopped by the addition of Dole's reagent (2-propanol:hexane:H_2_SO_4_ = 80∶20∶1), followed by heptane and water, and then vortexing. The upper layer was removed and the lower (aqueous) phase was washed three times with heptane. The radioactivity of the aqueous phase was evaluated with a liquid scintillation counter. The DNA concentrations in solubilized cells were measured with Hoechst dye 33258 (Dojindo Laboratories, Kumamoto, Japan). The ACS activity was normalized to DNA content.

### Western Blot Analysis

HASMC were prepared in lysis buffer (50 mM HEPES, 150 mM NaCl, 1 mM EDTA, 1 mM EGTA, 10% Glycerol, 1% Triton X-100) for measurement of intracellular ACSL3 protein. On the other hand, nuclear extracts were prepared using the NE-PER® Nuclear and Cytoplasmic Extraction Reagent Kit (PIERCE Biotechnology, Rockford, IL) for nuclear phospho-NF-κB (p65). Each sample was resolved on SDS-PAGE gels followed by transfer to nitrocellulose membranes. The membranes were blocked for 1 hour in blocking buffer at room temperature and then incubated for 1 hour or overnight with the appropriate primary antibodies: anti-ACSL3, anti-β-actin (Santa Cruz Biotechnology, Santa Cruz, CA), anti-ACSL1, anti-ACSL4 (Abcam, Cambridge, UK) anti-phospho-NF-κB (p65) or anti-lamin A/C (Cell Signaling Technology, Boston, MA). The membranes were washed and incubated with anti-goat (for ACSL3, R&D SYSTEMS, Minneapolis, MN), anti-mouse (for β-actin, Cell Signaling Technology) and anti-rabbit (for ACSL1, ACSL4, phospho-NF-κB (p65) and lamin A/C, Cell Signaling Technology) IgG HRP-conjugated secondary antibodies diluted to 1∶10000 for ACSL1, ACSL3 and ACSL4, and 1∶7500 for others. After three time-washes, the membranes were incubated with ECL Plus Western Blotting Detection Reagents (GE Healthcare Life Science, Piscataway, NJ). Chemiluminescent signals were visualized using X-ray film.

### Overexpression of ACSL3

The full length cDNA for human ACSL3 was fused to mammalian expression vector (pCI-neo) and cloned into the adenovirus vector to yield Ad-ACSL3. HASMC were infected with control adenovirus (Ad-LacZ) or Ad-ACSL3 at a multiplicity of infection of 20. HASMC were stimulated with 250 µM of PA for 1 day. The measurement of mRNA expression was performed as noted above.

### NF-κB Luciferase Assay

For NF-κB luciferase assay, firefly luciferase plasmid containing NF-κB binding element (pNF-κB-Luc) (Agilent Technologies, Santa Clara, CA) was transfected into HASMC using Lipofectamine™ LTX with Plus™ Reagent (Invitrogen) after several hours after seeding. Subsequently HASMC were stimulated with 250 µM PA in the presence or absence of 15 µM EPA or 2 µM of triacsin C for 1 day. After incubation, cells were harvested and luciferase assays were performed using Dual-Luciferase® Reporter Assay System (Promega, Madison, WI) according to the manufacturer’s protocol. The luciferase luminescence was normalized by internal control renilla plasmid.

### Calcium Deposition

HASMC were centrifuged (200×g, 1 min), washed twice with PBS, and incubated overnight at 4°C in 0.6 M HCl. The calcium concentrations in HCl supernatants were determined by the methylxylenol blue method using the Calcium E-test Wako (Wako Pure Chemical Industries, Osaka, Japan). After decalcification, the cells were washed three times with PBS and solubilized with 0.1 M NaOH containing 0.1% (w/v) SDS. The protein concentrations in solubilized cells were measured with the BCA Protein Assay Kit (PIERCE Biotechnology). The calcium content of the cell layer was normalized to protein content.

### Detection of Cellular Calcium Deposition

HASMC were stimulated with 1 mM PA in the presence or absence of 15 µM EPA for 1 day. After incubation, cells were fixed with methanol. After fixation, calcium deposition was detected using Von Kossa Method for Calcium Kit (Polysciences, Warrington, PA) according to the manufacturer’s protocol.

### Analysis of Caspase Activity

HASMC were centrifuged (200×g, 1 min), washed twice with PBS, and caspase activities were determined using the Caspase-Glo® 3/7 Assay (Promega) according to the manufacturer’s protocol. The protein concentrations in cell lysates homogenized with M-PER® (PIERCE Biotechnology) in another plate were measured with the BCA Protein Assay Kit. Caspase-3/7 activity was corrected for mean protein content in each group.

### Apoptosis Assay

HASMC were centrifuged (200×g, 1 min), washed twice with PBS, and apoptosis assay was performed using Cell Death Detection ELISA (Roche Diagnostics, Mannheim, Germany) according to the manufacturer’s protocol.

### Immunohistochemistry

The human carotid arteries were obtained by autopsy at Gunma University Hospital. This protocol was approved by the Institutional Review Board at Gunma University Hospital, and written informed consent was obtained from patients’ families. Specimens were fixed with 4% formaldehyde and embedded in paraffin. For confocal microscopy, sections were incubated with antibodies against ACSL3, smooth muscle (SM) α-actin, CD68 and Msx2. These antibodies were from Santa Cruz Biotechnology, California, USA. For double-immunofluorescence staining samples were incubated with Cy3- and fluorescein isothiocyanate (FITC)-conjugated secondary antibodies (Sigma) for 1 hour at room temperature. Nuclear staining was performed with 4′,6′-diamidino-2-phenylindole (DAPI). Immunohistochemical staining of sections was performed using the CSA kit (DAKO, Glostrup, Denmark) according to the manufacturer’s protocol.

### Statistical Analysis

All results are presented as the mean ± S.E. Statistical comparisons were performed by Dunnett’s tests or Tukey-Kramer’s tests. Values of p<0.05 were considered significant.

## Results

### PA Induces Osteoblastic Differentiation of VSMC

**Figure 1:**
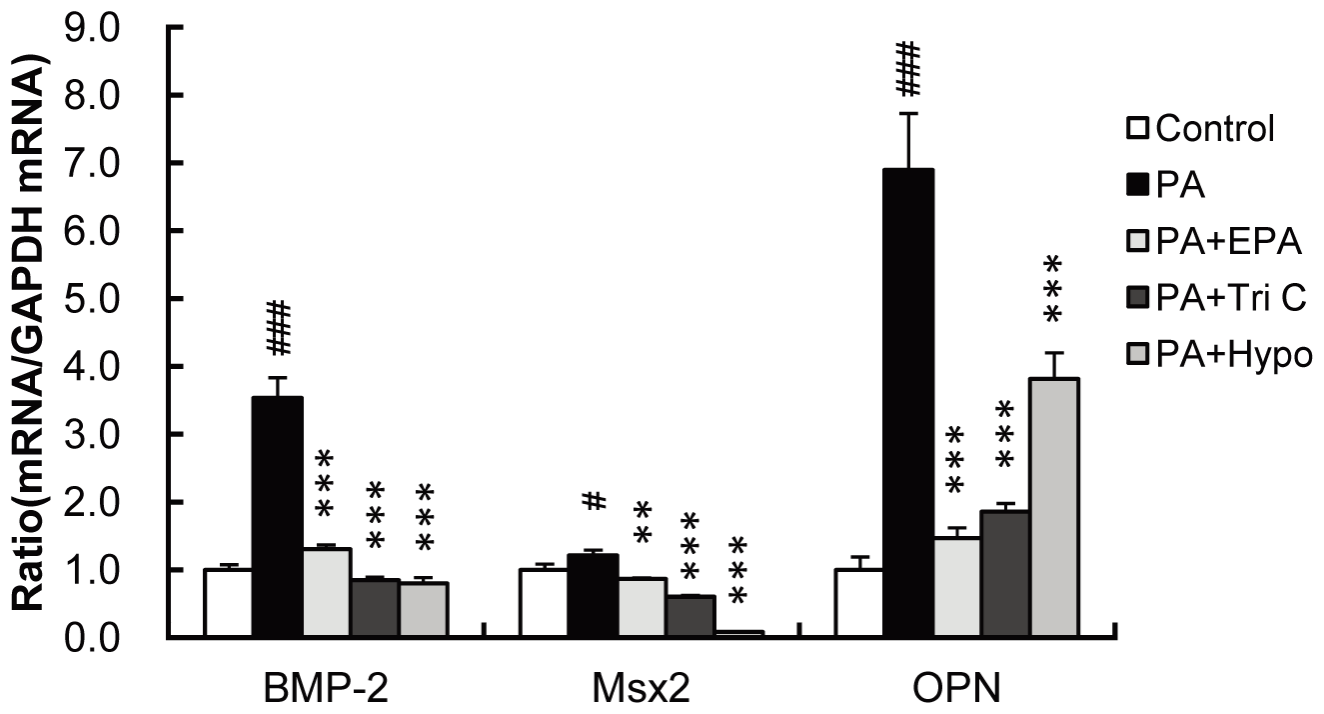
Effects of PA, EPA and inhibitors of ACS and NF-κB on the expression of bone-related genes in HASMC. HASMC were treated with PA (250 µM) for 1 day in the presence or absence of EPA (15 µM), triacsin C (Tri C; 2 µM) or hypoestoxide (Hypo; 100 µM). The expression of BMP-2, Msx2 and osteopontin (OPN) mRNAs was determined by real-time PCR and normalized to GAPDH. The control values are shown as 1.0. All values expressed by arbitrary unit (AU) are presented as the mean ± S.E. (n = 4). #p<0.05, ###p<0.001 vs. control, **p<0.01, ***p<0.001 vs. PA.

In a first step, we studied whether PA affected the expression of the genes coding for bone-related proteins in HASMC, a primary culture of VSMC derived from human aorta, by measuring the mRNA levels by real-time PCR. The mRNA levels for the embryonic bone differentiation factor BMP-2, osteoblastic transcription factor Msx2 and bone matrix protein osteopontin, were significantly elevated by PA treatment (Figure 1). Such an effect of PA on the gene expression of bone-related proteins was substantially inhibited by EPA (15 µM), triacsin C (2 µM, ACS inhibitor) and hypoestoxide (100 µM, IKK inhibitor). These findings suggest that PA induces the expression of bone-related genes in HASMC, and that activation of ACS and NF-κB mediates this effect.

### PA Increases ACSL3 mRNA and Protein Levels and ACSL3 Promoter Activity

**Figure 2:**
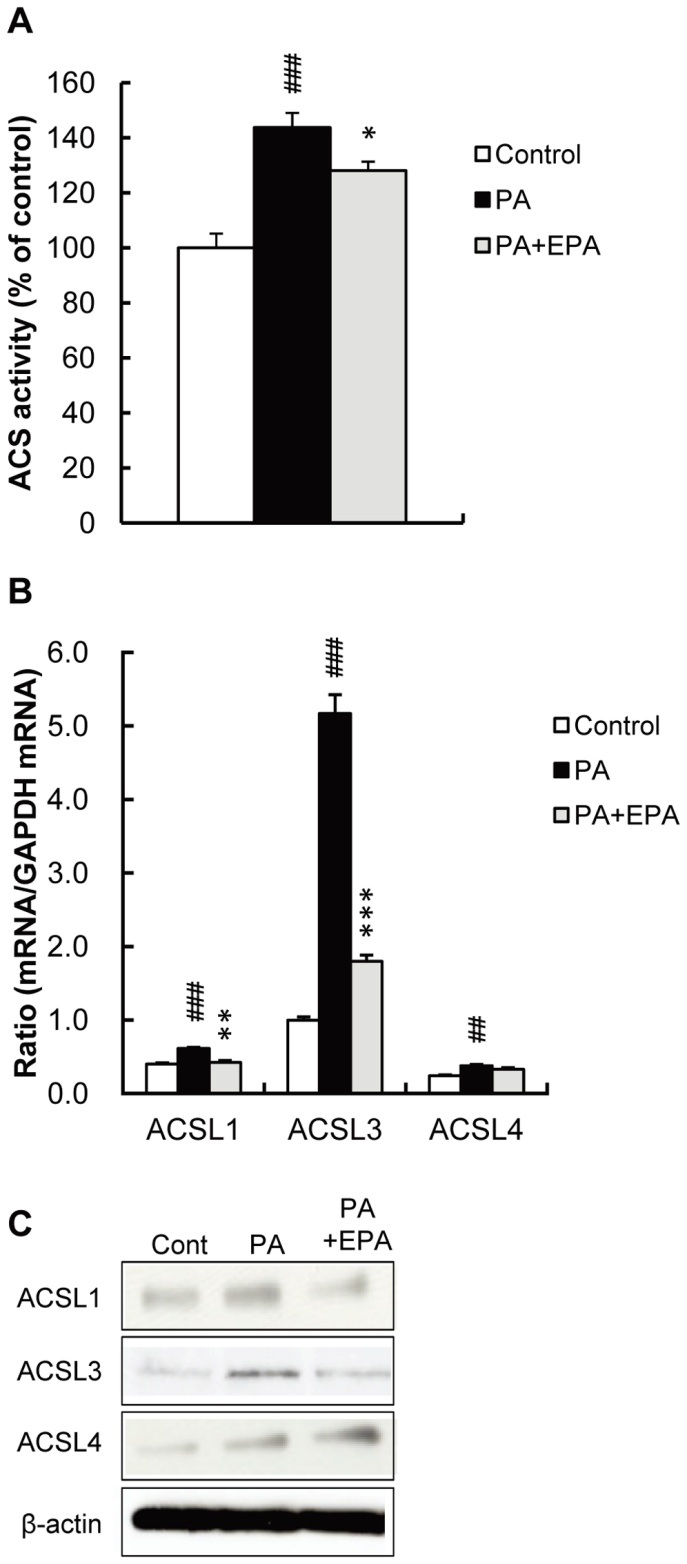
PA increases ACS activity and ACSL3 mRNA level in HASMC. HASMC were treated with PA (250 µM) for 1 day in the presence or absence of EPA (15 µM). A, ACS activity in cell homogenates was determined as described in Materials and Methods. B, Expression of ACSL1, 3 and 4 mRNAs was determined by real-time PCR and normalized to GAPDH. C, The expression of ACSL1, 3 and 4 protein was determined by Western blot analysis. All values expressed by AU are presented as the mean ± S.E. (n = 3–9). ##p<0.01, ###p<0.001 vs. control, *p<0.05, **p<0.01, ***p<0.001 vs. PA (except for C).

To determine the role of ACS in PA-induced osteoblastic differentiation in HASMC, we next measured ACS activity. ACS activity was significantly increased by PA, and EPA attenuated this response (Figure 2A). Among the long chain acyl-CoA synthetase subfamily, ACSL3 expression was most predominant, followed by ACSL1 and ACSL4 in HASMC (Figure 2B), and ACSL5 and ACSL6 were barely expressed (data not shown). Interestingly, expression of ACSL3 mRNA was robustly increased by PA (5.17±0.26-fold, p<0.001) while inductions of ACSL1 and ACSL4 were 1.53±0.04-fold (p<0.001) and 1.57±0.06-fold (p<0.01), respectively. Interestingly, PA induction of ACSL3 mRNA expression was largely inhibited by EPA. Western blot analysis confirmed the induction of ACS3 levels by PA and a prevention of this effect by EPA, whereas there were no measurable changes in ACSL1 and ACSL4 (Figure 2C). These results suggest that a prevention of PA-induced osteoblastic differentiation by EPA is associated with the inhibition of ACSL3 expression.

### PA Induces ACSL3 Expression Prior to the Induction of the Osteoblastic Gene Expression

**Figure 3:**
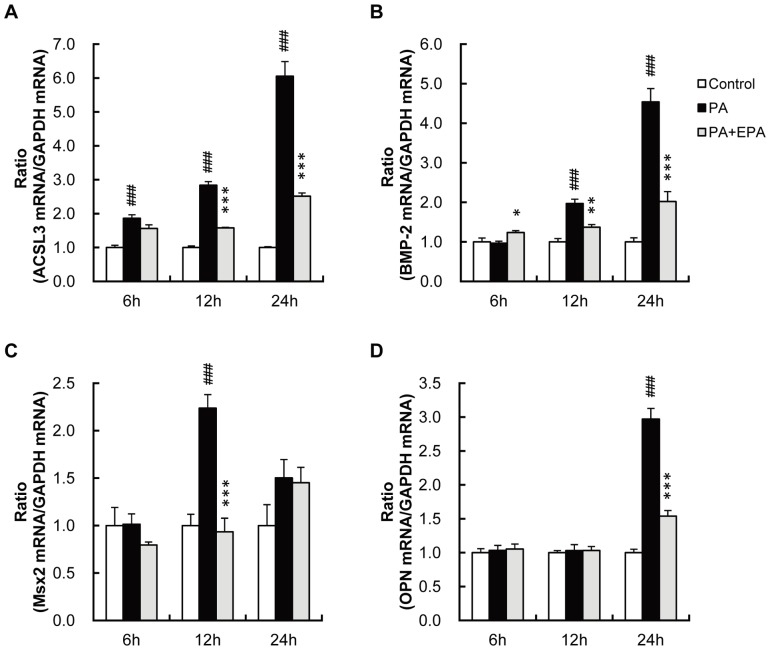
PA increases mRNA level for ACSL3 prior to that for osteoblastic genes in HASMC. HASMC were treated with PA (250 µM) with or without EPA (15 µM). Expressions of ACSL3 (A), BMP-2 (B), Msx2 (C) and OPN (D). The mRNA levels at 6, 12 and 24 hours after treatment with PA and EPA were determined by real-time PCR and normalized to GAPDH. The control values are shown as 1.0. All values expressed by AU are presented as the mean ± S.E. (n = 4). ###p<0.001 vs. control, *p<0.05, **p<0.01, ***p<0.001 vs. PA.

We examined the mRNA levels of ACSL3 and osteoblastic genes at 6, 12 and 24 hours after treatment with PA. Induction of ACSL3 was readily detected at 6 hours, and preceded the increase in expression of osteoblastic genes such as BMP-2, Msx2 and osteopontin (Figures 3A–3D): an increase in BMP-2 and Msx2 was observed at 12 hours, while measurable increase in osteopontin mRNA level was not detected until 24 hours. These findings conform to our hypothesis that PA-induced ACSL3 expression plays a causative role in PA-induced osteoblastic gene expression in VSMC.

### ACSL3 Regulates PA-induced Osteoblastic Gene Expression

**Figure 4:**
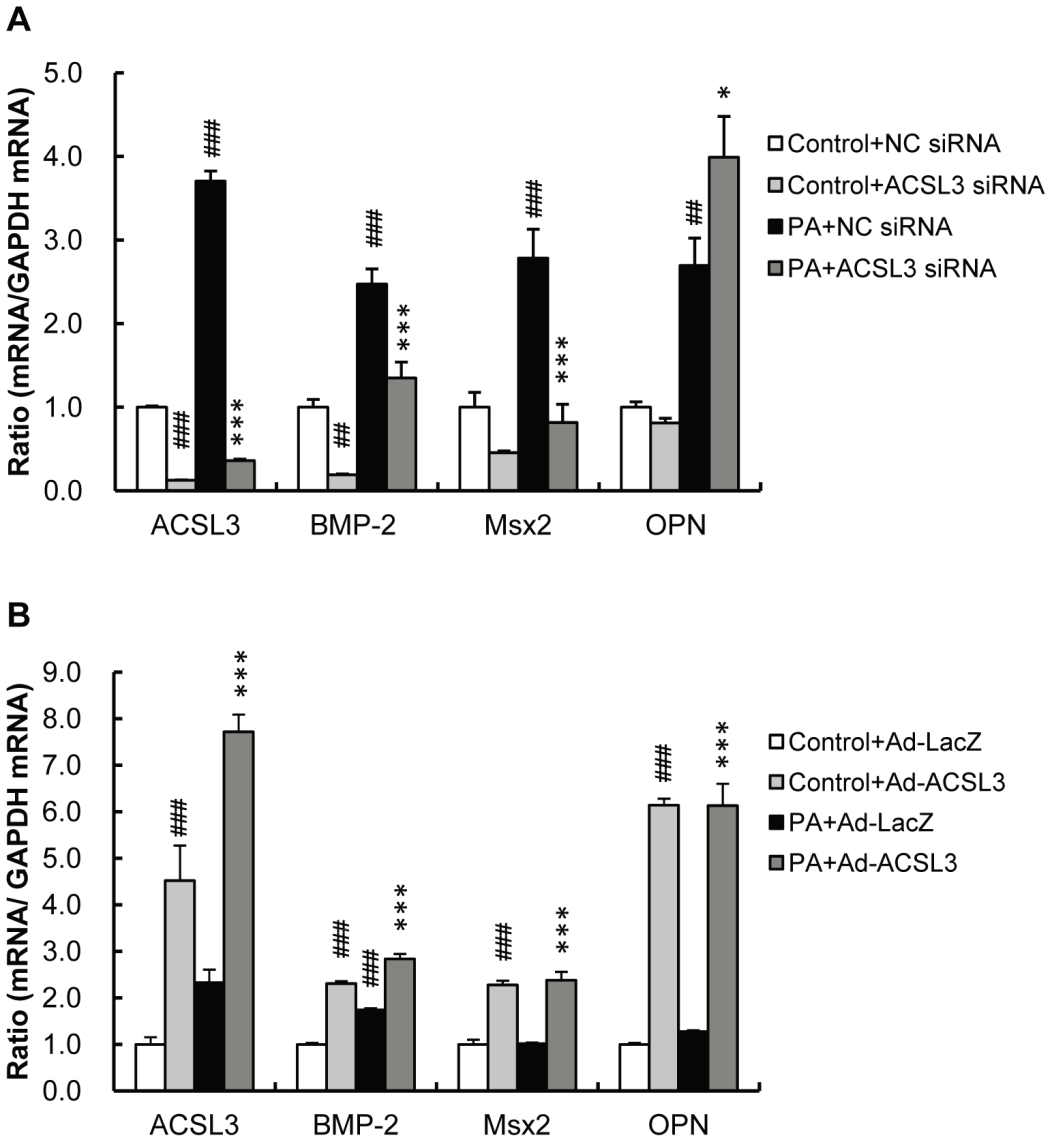
Knock-down and overexpression of ACSL3 attenuates and augments PA-induced expression of osteoblastic genes in HASMC. A, HASMC were transfected with either ACSL3 siRNA or nonsilencing negative control (NC) siRNA, and then cells were treated with PA (250 µM) for 1 day. Expressions of ACSL3, BMP-2, Msx2 and OPN mRNA were determined by real-time PCR and normalized to GAPDH. B, HASMC were infected with Ad-LacZ or Ad-ACSL3 and then treated with PA (250 µM) for 1 day. Expression of ACSL3, BMP-2, Msx2 and OPN mRNAs were determined by real-time PCR and normalized to GAPDH. The control values are shown as 1.0. All values expressed by AU are presented as the mean ± S.E. (n = 4–6). ##p<0.01, ###p<0.001 vs. control+NC siRNA or control+Ad-LacZ, *p<0.05, ***p<0.001 vs. PA+NC siRNA or PA+Ad-LacZ.

To directly test whether an induction of ACSL3 by PA plays an indispensable role in PA-induced osteoblastic differentiation of VSMC, HASMC were transfected with siRNA of ACSL3 at 1 day prior to PA treatment. ACSL3 mRNA expression was successfully reduced below 90% by ACSL3 siRNA compared with negative control (NC) siRNA. Importantly, knock-down of ACSL3 expression significantly attenuated PA-induced expression of BMP-2 and Msx2 (Figure 4A). On the other hand, an expression of osteopontin mRNA was not inhibited but rather induced by ACSL3 siRNA.

To confirm the role of ACSL3 in PA-induced osteoblastic differentiation of VSMC, HASMC were infected with adenovirus expressing either ACSL3 or lacZ, and the expression of osteoblastic genes was measured by real-time PCR. Results showed that overexpression of ACSL3 *per se* enhanced the expression of osteoblastic genes but it did not further enhance the expression of these genes in the presence of PA (Figure 4B). Taken together, these findings suggest that an induction of ACSL3 is necessary and sufficient for PA-induced osteoblastic differentiation of HASMC.

### PA-Induced Activation of NF-κB (p65) is Mediated by the ACSL3-dependent Pathway

**Figure 5:**
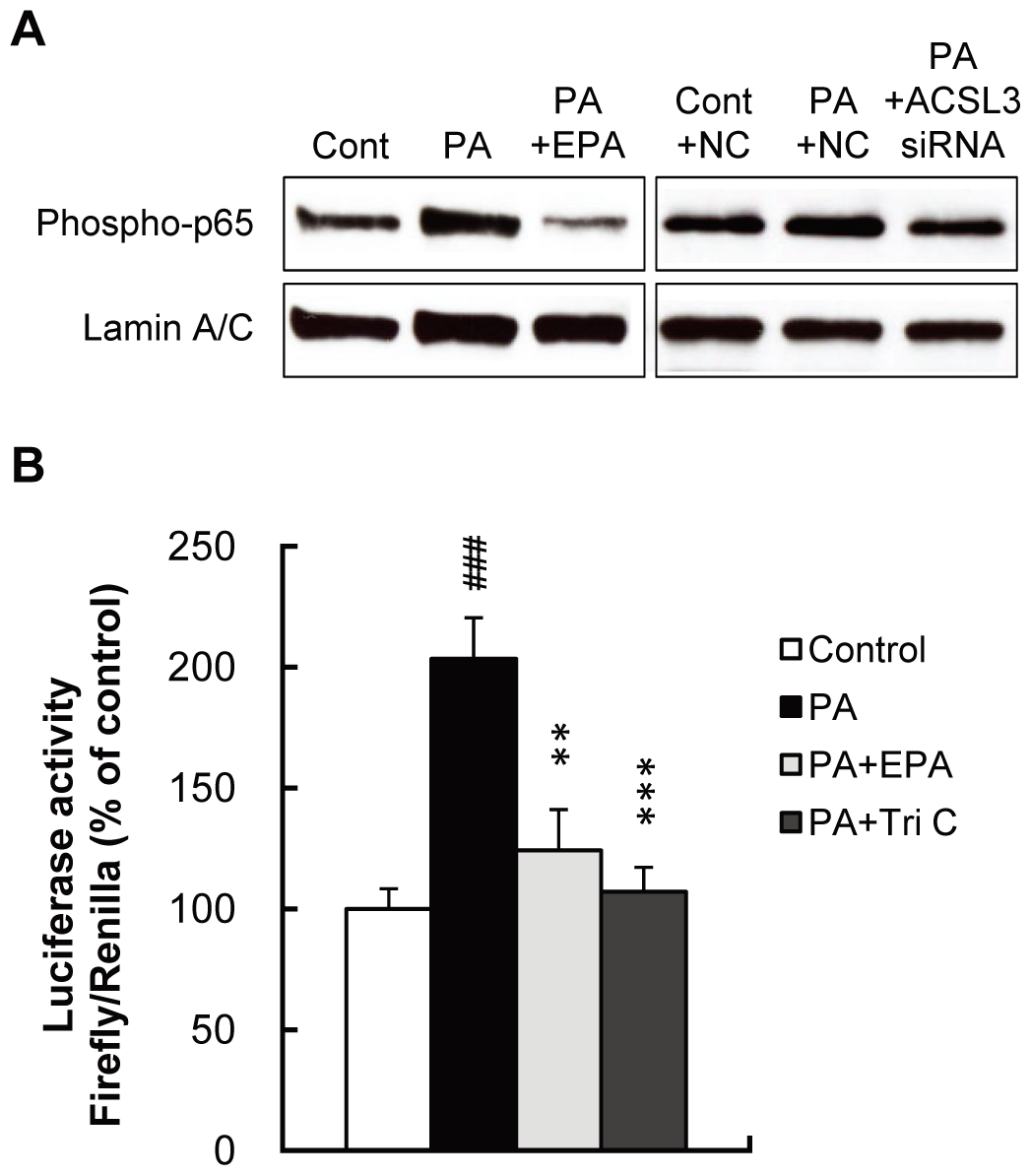
PA induces NF-κB activation. A, HASMC were treated with PA (250 µM) for 1 day with or without EPA (15 µM). Either ACSL3 siRNA or nonsilencing control (NC) siRNA was transfected before PA treatment. Expression of nuclear phospho-NF-κB (p65) protein was determined by Western blot analysis. B, HASMC were transfected with pNF-κB-Luc and then treated with PA (250 µM) for 1 day with or without EPA (15 µM) or Tri C (2 µM). The luciferase activity of the NF-κB reporter was measured according to the manufacturer’s protocol. All values expressed by AU are presented as the mean ± S.E. (n = 6). ###p<0.001 vs. control, **p<0.01, ***p<0.001 vs. PA.

Based on the observation that hypoestoxide (100 µM, IKK inhibitor) prevented the PA-induced expression of osteoblastic genes (Figure 1), we next examined the effects of PA or PA plus EPA on the phosphorylation of NF-κB (p65) prepared from HASMC (Figure 5A). Nuclear phospho-NF-κB (p65) levels were elevated by PA compared to control, and this was prevented by EPA. Furthermore, siRNA-mediated knock-down of ACSL3 but not nonsilencing NC siRNA attenuated an increase in phospho-NF-κB (p65) levels by PA. In addition, luciferase assay showed that PA increased transcriptional activation of pNF-κB-Luc, a plasmid containing NF-κB binding elements and this effect was significantly prevented by EPA and triacsin C (Figure 5B). These findings suggest that ACSL3 regulates an intracellular event upstream of NF-κB-induced signaling pathway.

### PA Induces Calcium Deposition in VSMC

**Figure 6:**
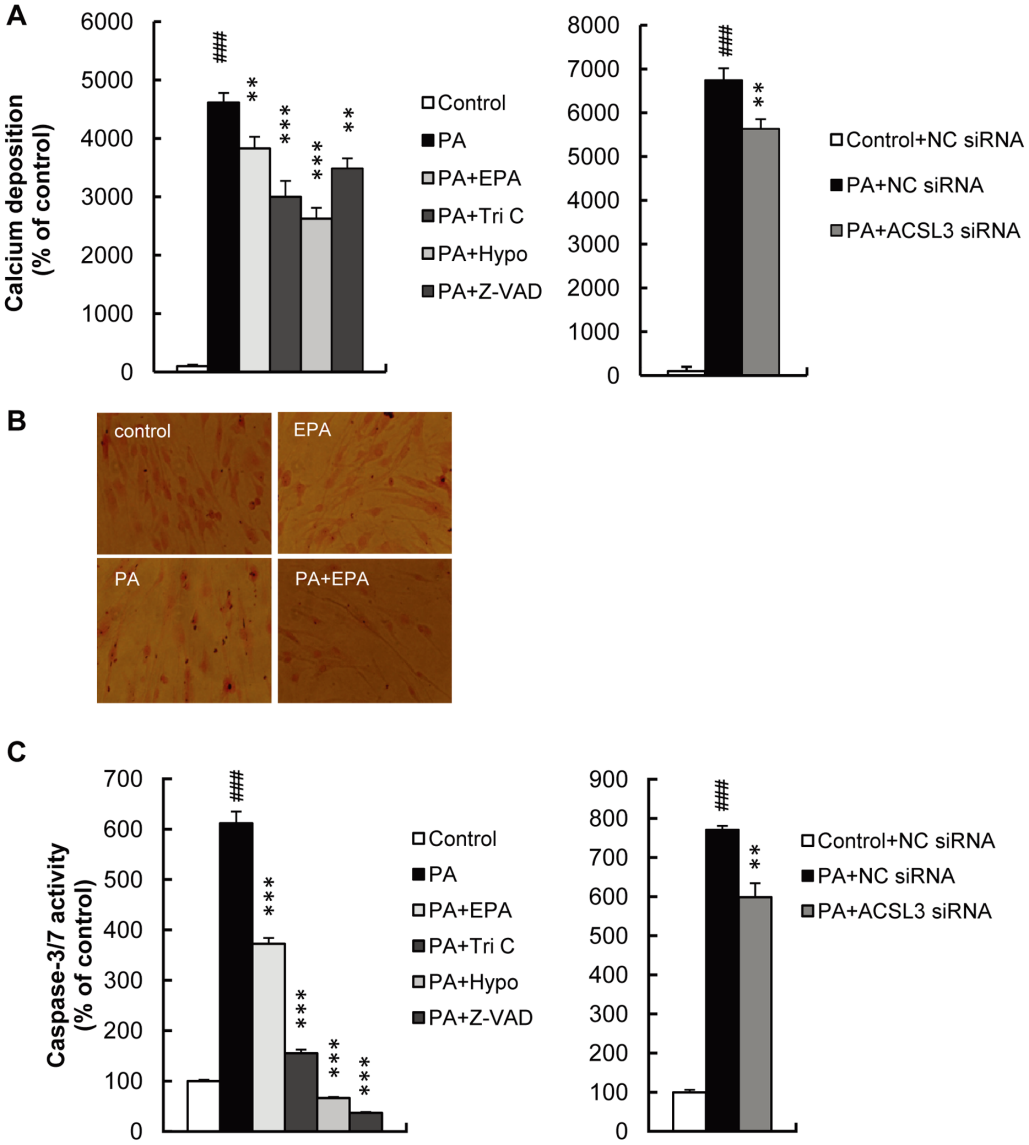
High-concentration PA induced calcium deposition and caspase activation in HASMC. HASMC were treated with PA (1 mM) for 1 day with or without EPA (15 µM), Tri C (2 µM), Hypo (100 µM) or Z-VAD-FMK (Z-VAD; 100 µM). ACSL3 siRNA were transfected before PA treatment. A, Calcium deposition was determined by the methylxylenol blue method and normalized to protein content. B, Calcium deposition was detected using von Kossa-staining. C, Caspase-3/7 activity was determined as described in Materials and Methods, and normalized to protein content. All values are presented as the mean ± S.E. (n = 3–14). ###p<0.001 vs. control or control+NC siRNA, **p<0.01, ***p<0.001 vs. PA or PA+NC siRNA.

To test whether PA induces calcium deposition, HASMC were exposed to a high concentration of PA (1 mM) for 24 hours. As shown in Figure 6A, PA significantly induced calcium deposition. Triacsin C (2 µM), hypoestoxide (100 µM), Z-VAD-FMK (caspase inhibitor, 100 µM) and ACSL3 siRNA as well as EPA (15 µM) prevented PA-induced calcium deposition. To confirm calcium deposition, we performed von Kossa-staining and found more calcium nodules in PA-treated HASMC. Consistent with the aforementioned results, EPA inhibited PA-induced calcium deposition (Figure 6B). Among various isoforms of ACSLs including ACSL1 and ACSL3-ACSL6, ACSL3 mRNA was the most abundant isoform in HASMC and was the most robustly induced by PA in HASMC (data not shown). These findings suggest that PA induces calcium deposition in VSMC through pathway involving ACSL3- and NF-κB.

### PA Induces Caspase Activation in VSMC

To examine whether apoptosis is associated with PA-induced calcium deposition, we measured caspase activity in HASMC stimulated with high concentrations of PA. One mM of PA increased caspase-3/7 activity in HASMC by 6.12±0.23 (p<0.001) or 7.72±0.09-fold (p<0.001) (Figure 6C). Such an increase in caspase-3/7 activity was prevented by EPA, inhibitors of ACS and IKK, and ACSL3 siRNA. These findings indicate that PA-induced calcium deposition is mediated in part by apoptosis.

### Expression of ACSL3 in Normal and Calcified Human Carotid Arteries

**Figure 7:**
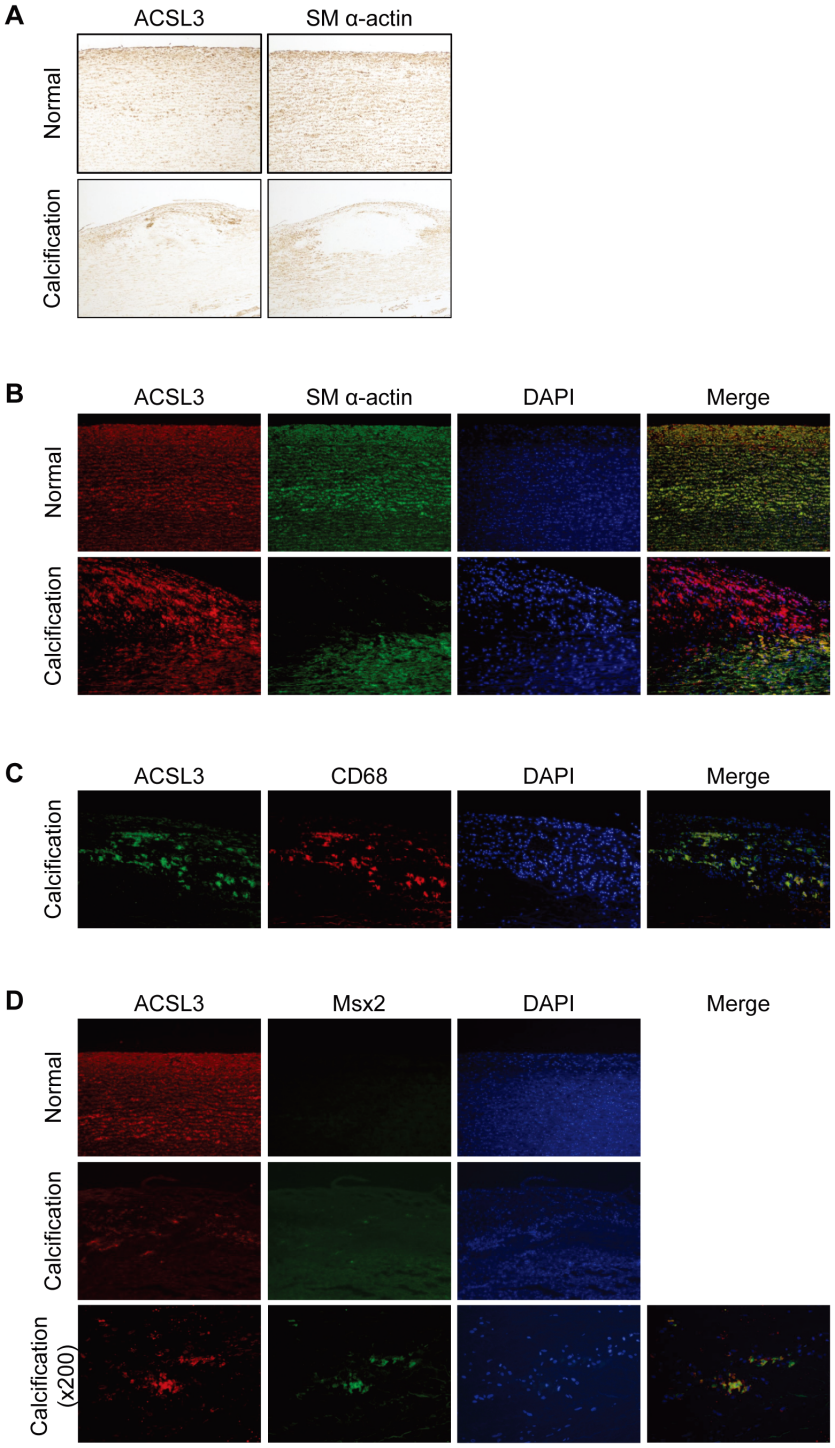
The expression of ACSL3 in normal and calcified human carotid artery. A, Immunohistochemistry for ACSL3 and SM α-actin in normal (upper panel) and calcified human carotid artery (lower panel). Magnification ×100. B to D, Immunofluorescent labeling for ACSL3 (red or green), (B) SM α-actin (green), (C) CD68 (red) or (D) Msx2 (green) and DAPI (blue) in normal and calcified human carotid artery. Magnification ×100 and ×200 (only calcified human carotid artery in D).

To corroborate that ACSL3 is expressed in complex calcifying atherosclerotic lesions *in vivo*, we performed immunohistochemical analysis on human carotid arteries. As shown in Figure 7A, normal arteries showed uniform staining for ACSL3 in cells that are stained positive for SM α-actin, indicating that ACSL3 is expressed in VSMC. In contrast, calcified arteries showed intense ACSL3 immunoreactivity in both SM α-actin-positive and SM α-actin-negative cells (Figure 7B). Immunofluorescence images of calcifying atherosclerotic lesion depicted the colocalization of ACSL3 with macrophage CD68 immunoreactivity (Figures 7C). In addition, Msx2 expression was observed in the some of the ACSL3-positive cells in atherosclerotic lesion (Figure 7D). These observations indicate that ASCL3 is expressed in VSMC as well as in macrophages of human calcifying atherosclerotic lesions which express Msx2.

## Discussion

**Figure 8:**
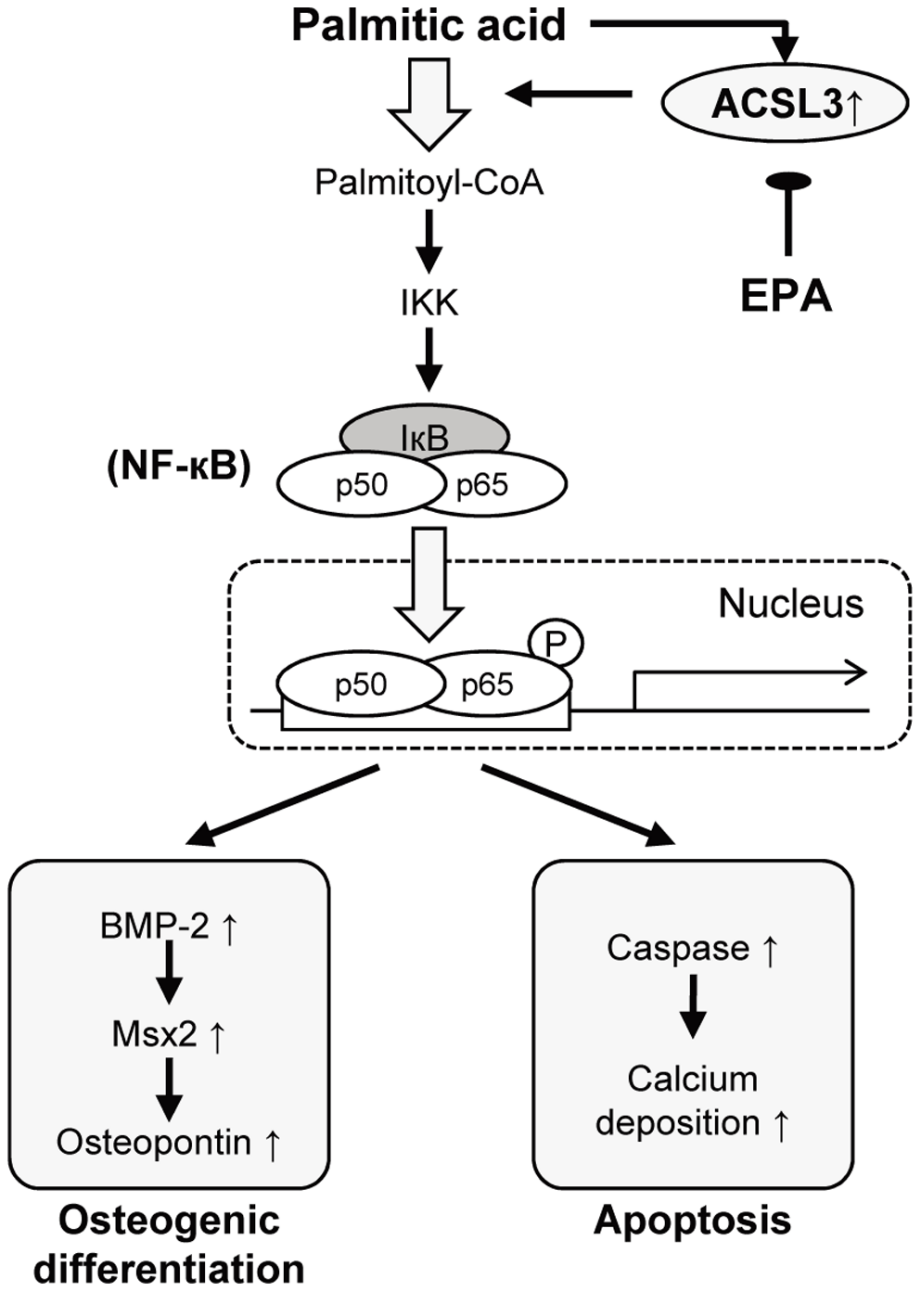
Schematic diagram of possible effects of PA and EPA on osteoblastic differentiation and calcium deposition in HASMC.

In the present study, we provided four lines of independent evidence indicating that PA induces osteoblastic differentiation and mineralization through ACSL3 activation. First, we showed that PA selectively induced ACSL3 expression among the ASCL family members in HASMC. Second, siRNA-mediated knock-down of ACSL3 expression as well as triacsin C, a pharmacological inhibitor of ACS, effectively prevented the PA-induced expression of the genes for bone-related proteins and calcium deposition. Third, adenovirus-mediated overexpression of ACSL3 enhanced osteoblastic differentiation of HASMC. Forth, immunohistochemistry of the human calcifying atherosclerotic plaque in carotid arteries revealed that ACSL3 is expressed in SMC as well as in macrophages infiltrated into calcifying lesion. Based on these findings, we propose the working model of PA-induced vascular calcification in Figure 8, illustrating that ACSL3 plays a crucial role in this process.

There are precedent studies by Miyazaki and his colleagues demonstrating that *de novo* synthesized and exogenously applied saturated fatty acids SA induced osteoblastic differentiation and mineralization in CVC and VSMC, respectively [11], [12]. They showed that ACS inhibitor prevented the osteoblastic differentiation induced by SA and stearoyl-CoA desaturase inhibitor promoted osteoblastic differentiation, and proposed that stearoyl-CoA or its metabolite lead to vascular calcification. It should be noted that SA induced mineralization and ALP expression to a greater extent than PA in CVC [11]. In contrast, our results point to a role for PA in osteoblastic differentiation and mineralization of HASMC. Considering that PA and SA may have distinct influence on cell function, we compared the effects between PA (250 µM) and SA (250 µM) on expression of osteogenic markers and induction of apoptosis. Results indicated that PA and SA exerted comparable pro-osteoblastic and pro-calcific effects in HASMC (data not shown). Further studies will be required to elucidate the differential roles in vascular calcification between PA and SA since several genes involved in apoptosis are differentially regulated between PA and SA as determined by microarray analysis [31].

Our study provided the evidence that ACSL3 exerts a crucial role in mediating pro-calcifying effects of PA. The ACS families can be divided into five subfamilies (short-chain, medium-chain, long-chain, bubblegum and very long-chain) which differ in substrate specificity depending on chain length. Both PA and EPA are activated by the ACSL subfamily. Five different isoforms of ACSL subfamily (ACSL1, ACSL3, ACSL4, ACSL5 and ACSL6) have been identified in humans [32]. Our data showed that among these isoforms, expression of ACSL3 was predominant in HASMC, and was highly induced by PA, while an induction of ACSL1 and ACSL4 was minimal, if any, by PA.

The differential effects of EPA on the expression of osteoblastic markers with or without PA deserve additional discussion. Although siRNA for ACSL3 inhibited osteoblastic gene expression both in the presence and absence of PA, EPA had no effects on osteoblastic gene expression in the absence of PA (data not shown). These results may provide clues to the mechanisms by which EPA inhibits ACSL3-mediated osteoblastic differentiation. We reason that EPA exerts its inhibitory effects on PA-induced ACSL3 expression but not on the expression and/or activity of pre-existing ACS. This assumption may also explain the data showing that an inhibition of PA-induced osteoblastic gene expression by EPA was remarkable, whereas EPA modestly inhibited ACS activity (Figures 1 and 2). Furthermore, consistent with this possibility, we observed that EPA effectively inhibited PA-induced promoter activity of the human ACSL3 gene without affecting basal promoter activity as determined by transient transfection assays of the luciferase reporter genes (data not shown). However, another possibility that EPA inhibits osteoblastic gene expression through ACSL3-independent as well as ASCL3-dependent mechanisms cannot be excluded. Further research to explore the underlying mechanisms regulating ACSL3 expression is now in progress.

Immunohistochemistry of human carotid arteries showed that ACSL3 is expressed in VSMC as determined by the colocalization with SM α-actin in both normal and atherosclerotic calcifying lesion. Consistent with the previous reports [33], [34], SM α-actin expression was down-regulated in the calcifying lesion. Interestingly, ACSL3 expression was abundantly expressed in cells that stained positively with CD68 antibody in calcifying artery, implying that macrophages express ACSL3 in calcifying artery. Accordingly, ACSL3 expression levels were varied dependent upon cellular composition of the arterial wall. It is reported that macrophages contribute to vascular calcification *via* expressing elastase such as matrix metalloproteinase (MMP)-2 and MMP-9 [16]. Although our data seem to suggest some role of macrophage ACSL3 in the development of vascular calcification, further investigation is required to clarify that.

One of the salient findings is that an increase in PA-induced osteoblastic gene expression was prevented by EPA at least in part *via* suppression of ACSL3 expression. Abedin et al. [17] showed that EPA and docosahexaenoic acid inhibit alkaline phosphatase activity and mineralization of CVC, which spontaneously undergo osteoblastic differentiation and mineralization, *via* peroxisome proliferator-activated receptor (PPAR)-γ and activation of p38-MAPK. In our study, PPAR-γ agonists and p38-MAPK inhibitor did not affect the osteoblastic gene expression regulated by PA and EPA in HASMC (data not shown).

In parallel, IKK inhibitor prevented PA-induced expression of osteoblastic differentiation. Moreover, PA elevated phosphorylation of nuclear NF-κB (p65) protein and transcriptional activity of NF-κB. These findings show that the osteoblastic differentiation induced by PA is mediated by the IKK-NF-κB pathway, and are consistent with the previous report indicating that expression of BMP-2 is up-regulated through NF-κB signaling [35]. It is noteworthy that siRNA for ACSL3 inhibits PA-induced phosphorylation of p65 subunit of NF-κB, and ACS inhibitor prevents PA-induced transcriptional activity. These results indicate that ACSL3 acts upstream of the NF-κB pathway and palmitoyl-CoA or further metabolites of palmitoyl-CoA activates NF-κB signaling pathway through the activation of ACSL3.

Previous studies described the role of NF-κB in PA-induced gene expression, in which PA induces IL-6 gene expression in human primary skeletal muscle cells through a rapid and chronic activation of NF-κB [36]. In that study, triacsin C did not prevent IL-6 induction by PA but rather enhanced IL-6 mRNA expression, implying that the free, non-metabolized-PA is responsible for the IL-6 gene activation. Taken together, these data suggest that the role of ACSL in PA-induced gene expression differs dependent upon the target genes. This assumption may explain at least in part an induction rather than inhibition of osteopontin gene expression in HASMC treated with ACSL3 siRNA (Figure 4A).

Apoptosis followed by calcium deposition is another mechanism of vascular calcification. Apoptotic bodies derived from vascular smooth muscle cells act as cores of calcium deposition similar to matrix vesicles in osteoblasts and chondrocytes, and thus contribute to vascular calcification [37], [38]. In the present study, 1 mM PA sufficiently induced apoptosis as indicated by an induction of caspase-3/7 activity. We also detected an increase in apoptosis by PA as determined by the assays of fragmented DNA associated with nucleosomal histone (data not shown). Furthermore, caspase inhibitor prevented the PA-induced calcium deposition as well as apoptosis in HASMC, suggesting that apoptosis plays a causative role in PA-induced calcium deposition. Most notably, our study provides the evidence for the key role of ACSL3 and NF-κB activation in PA-induced apoptosis by showing that both caspase activation and calcium deposition were blunted by inhibitors of ACS, IKK and ACSL3 siRNA.

Potential limitations of the present study need to be considered. First, in Figure 4B, PA did not increase Msx2 and OPN mRNA levels in HASMC transduced with Ad-LacZ. These unexpected results may be due to potential nonspecific effects of LacZ infection that elevates basal levels of expression of the genes of interests, and masks the specific induction by PA given the ROS-mediated gene transcription by virus infection as suggested by Schwarz [39]. Second, we exposed HASMC to a higher concentration of PA (1 mM) to test the effects of PA on calcium deposition because more than several days are required for HASMC to undergo mineralization in response to 250 µM of PA. Such a long term culture was not feasible for the experiments using ACS inhibitor, NF-κB inhibitor and siRNA because these reagents may affect cell viability during culture. Our observation that EPA only partly inhibited PA-induced calcium deposition whereas it markedly inhibited PA-induced osteoblastic gene expression likely reflects the difference in PA concentration. Future study will be required to clarify this possibility. Another limitation is the immunohistochemistry of human calcifying carotid artery while we used VSMC derived from aorta in *in vitro* experiments. Potential difference in calcification process between elastic artery aorta and muscular artery carotid artery should be explored in further study.

In summary, this study demonstrate that PA induces mineralization of HASMC by increasing ACSL3 expression and by activating NF-κB signaling which mediate osteoblastic differentiation and apoptosis, respectively. EPA prevents initiation of mineralization by inhibiting PA-induced ACSL3 expression. Since vascular calcification is closely associated with cardiovascular morbidity and mortality, significant reduction in cardiovascular event rate shown in JELIS may be at least partly attributed to a prevention of vascular calcification by EPA [13]. Future clinical studies should be warranted to evaluate EPA on vascular calcification.
